# *RB1* Germline Variant Predisposing to a Rare Ovarian Germ Cell Tumor: A Case Report

**DOI:** 10.3389/fonc.2020.01467

**Published:** 2020-08-21

**Authors:** Elisa Gelli, Chiara Fallerini, Floriana Valentino, Annarita Giliberti, Francesca Castiglione, Lucrezia Laschi, Maria Palmieri, Alessandra Fabbiani, Rossella Tita, Maria Antonietta Mencarelli, Alessandra Renieri, Francesca Ariani

**Affiliations:** ^1^Medical Genetics, University of Siena, Siena, Italy; ^2^Histopathogy and Molecular Diagnostics, Careggi University Hospital Florence, Florence, Italy; ^3^Department of Health Sciences, University of Florence, Florence, Italy; ^4^Genetica Medica, Azienda Ospedaliera Universitaria Senese, Siena, Italy

**Keywords:** RB1, hypomorphic variant, yolk sac tumor, ovarian cancer, tumor predisposition

## Abstract

Malignant ovarian germ cell tumors (MOGCTs) are neoplasms of the ovary, of which, due to their rarity and heterogeneity, few is reported about genetic background and development. Here, we report a 18-years old patient diagnosed with an ovarian mixed germ cell tumor, without any previous history of malignancies, who has been treated with surgery and chemotherapy and died 4 years later due to peritoneal metastasis complications. Patient's blood DNA was screened for a panel of 52 cancer-related genes in order to identify predisposing aberrations to this rare cancer. The analysis discovered the uncharacterized c.2393G>A variant in *RB1*, the retinoblastoma gene, leading both to a missense change and a splicing perturbation of the *RB1* transcript. The variant was found to be hypomorphic, damaging the C-terminal domain with a partially impaired protein function. The variant is inherited from the unaffected mother. Due to an imprinting mechanism, the maternal allele is ~3-fold more expressed than the paternal one. The parent-of-origin effect combined with the hypomorphic impact of the variant determines a rescue of sufficient tumor-suppressor activity to prevent retinoblastoma development but can predispose to other cancers in the adult age. In order to understand the somatic events acting on the germline predisposition we used the NGS-liquid biopsy covering 77 cancer driver genes. Using this approach, we detected deleterious mutations in *TP53, SMAD4, FGFR3*, and *MSH2*, indicative of a dis-regulation of cell cycle and DNA repair mechanisms pathways. In conclusion, we have pinpointed for the first time that an *RB1* leaky variant, not leading to retinoblastoma because of its maternal origin, can predispose in adults to a very rare form of ovarian cancer and that the somatic disruption of few genes contributes to the tumor progression and aggressiveness.

## Introduction

Ovarian germ cell tumors (OGCTs) are histologically heterogeneous neoplasms arising from primitive germ cells. Most OGCTs are represented by mature cystic teratoma while, in the minority of cases, they present as malignant tumors (MOGCTs) including immature teratoma, dysgerminoma, yolk sac tumor, embryonal cell carcinoma, and choriocarcinoma. About 20–25% of ovarian neoplasms are OGCTs and only 5% of them are malignant. Globally MOGCTs represent the 2.6% of ovarian malignant neoplasm (1, 2). Due to their rarity, few is known about the genetics of MOGCTs: a germline *PTEN* frameshift variant was recently found in a pediatric patient carrying a mixed ovarian germ cell tumor associated with overgrowth. In other reports ovarian mixed germ cell tumors were associated with *BRCA1* or *BRCA2* mutations in familial contexts of hereditary breast and ovarian cancer syndrome (3–5).

*RB1* (#MIM 614041) is the first characterized and most studied tumor suppressor gene (6, 7). It is globally accepted that the encoded protein (pRB) mainly act as a tumor suppressor inhibiting the G1-S phase transition during the cell cycle by repressing the E2F transcription factors (8). Beyond this, many other protein interactions related to several functions other than cell cycle regulation have been discovered in the past years, making the pRB role more complex (9, 10). *RB1* inactivation is a quite frequent somatic event in many types of cancer, such as small cell lung cancer, soft tissue sarcoma, breast adenocarcinoma, bladder carcinoma (11), while germline inactivation has been related to predisposition to retinoblastoma (RB) and primary osteosarcoma following or not a previous retinoblastoma (12–14). Hereditary RB patients have a strongly increased risk for second primary malignancies in the adult age, including osteosarcoma, soft tissue sarcoma and melanoma (15–17). *RB1* is regulated by an imprinting mechanism which is responsible for a skewed gene expression in favor of the maternal allele, that is about 3 times more transcribed than the paternal one (18, 19). A parent-of-origin effect, due to this imprinting mechanism, has been proposed as an explanation for low penetrant pedigrees, in whom hypomorphic *RB1* variant inherited from the mother retain sufficient activity to suppress retinoblastoma development and carriers can show only benign lesions named retinoma or developing other types of cancer in adulthood (20–22).

Here, we employed an NGS-based strategy, combining the screening a panel of 52 cancer-related genes with a liquid biopsy approach to discover germline mutations predisposing to this rare cancer and investigate somatic mutations driving tumor progression.

## Methods

Patient samples were obtained at the Medical Genetics Unit (A.O.U.S, Siena, Italy) upon the signature of a written informed consent for both diagnostic and research purposes, in accordance with the Declaration of Helsinki. Detailed methodology is available in Supplementary Appendix.

### Case Description

**Figure 1:**
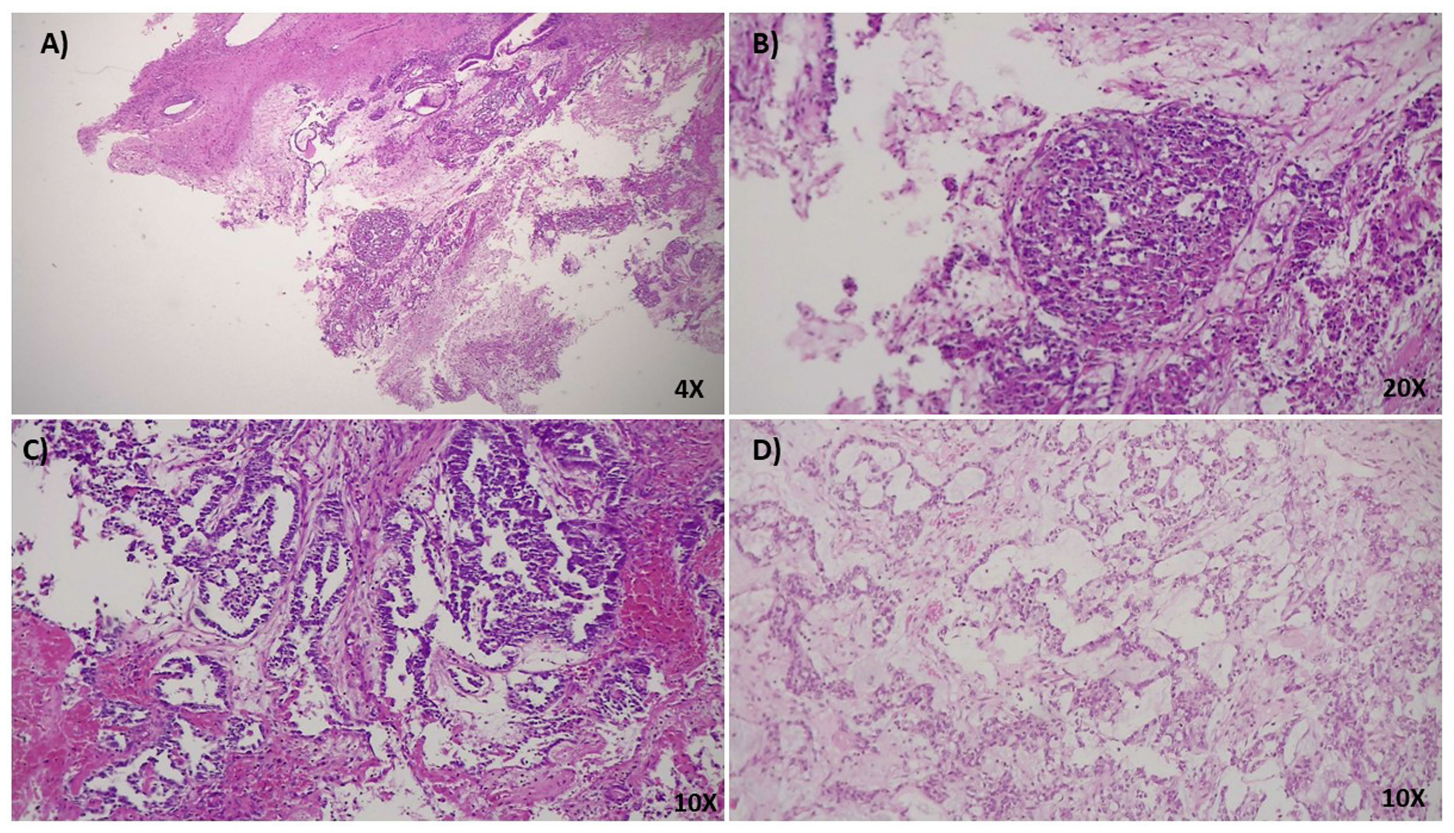
**(A)** 4x HPF, H&E stain. Ovarian cystic teratoma consisting of epidermoid elements and mature neuroectodermal tissue with focal morphological characteristics of a yolk sac tumor. **(B)** 20x HPF, H&E stain. At higher magnification, the limited and focal tumoral area shows typical characteristics of the hepatoid variant of yolk sac tumor. It can usually be found in young females, associated with increased levels of serum α-fetoprotein (AFP). To note, the presence of great polygonal cells with a well represented eosinophilic cytoplasm and abundant presence of hyaline bodies. **(C,D)** 10 x HPF, H&E stain. Teratoma shows abundant mature neuroectodermal tissue interspersed in the stroma with different and well-differentiated structures such as hair follicles. The presence of these elements indicates a high maturation level of the neoplasia.

**Figure 2:**
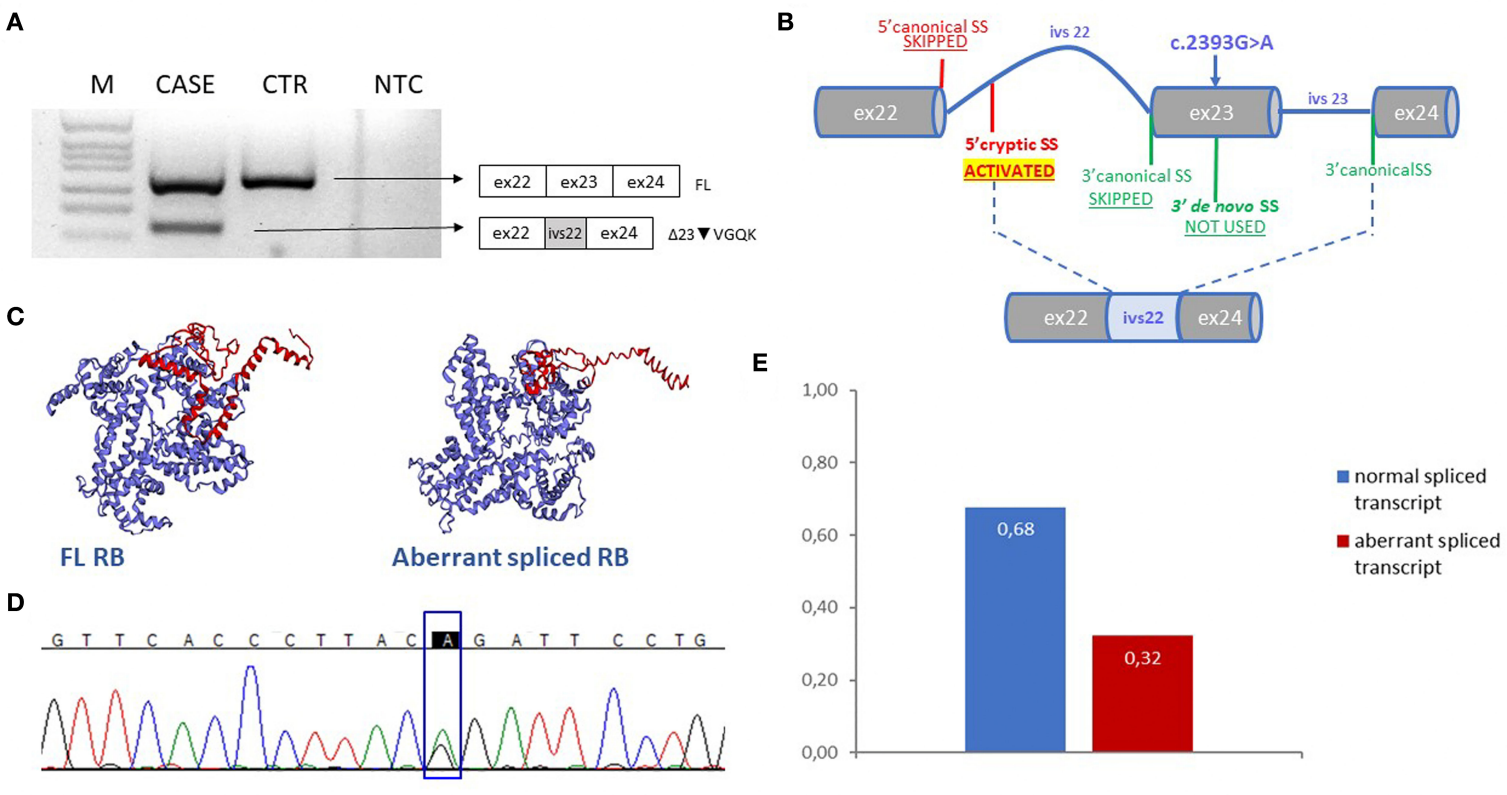
**(A)** Electrophoresis gel of the RT-PCR products of case and control sample with forward primer spanning exon 21-22 junction and reverse spanning exon 24–25 junction. Next to the gel the schemes corresponden to the amplified products. **(B)** Scheme explaining the mechanism of splicing of intron 22 and exon 23 in presence of the variant c.2393G>A; the figure shows the loss of the use of the canonical sites of exon 22 and 23, the generation of a *de novo* 3'SS and the activation of a cryptic 5' SS in intron 22 used with the canonical 3'SS of exon 24. This results in an aberrant exons junction with the incluion of part of intron 22 and the exclusion of exon 23 in the mature transcript. **(C)** Three-dimensional model of the FL and the aberrant spliced RB proteins predicted with EzMol, C-term domain is highlighted in red. **(D)** Electropherogram represents the sequence of the FL agarose band displayed in **(A)**, c.2393 position is indicated in the rectangle **(E)** The graph shows the result of the densitometric analysis of the CASE gel bands displayed in **(A)**; sum of the FL and aberrant bands was considered as the total and each value from both transcripts was compared to that. M, DNA molecular marker, CTR, healthy control sample, NTC, no-template control, FL, full length transcript.

Here, we report a case of a female patient who had a diagnosis of unilateral ovarian mixed germ cell tumor (mature teratoma and yolk sac tumor) at 18 years and who died at 22 years due to peritoneal and lymph node metastases. The available information on family history revealed no evidence of tumors. The cystic teratoma consisted of epidermoid elements, extensive areas of mature neuroectodermal tissue and a limited glandular area positive for AFP, with the typical histological features of yolk sac tumor (Figures 1A–D). The lesion was surgically removed and the patient was treated with estroprogestinic therapy until the appearance of metastases at 20 years. Bleomycin, etoposide and platinum (BEP) chemotherapy was then started (four cycles); 2 months later, after another surgical treatment, recurrent pelvic lesions compatible with mature teratoma were detected, so three cycles of combination chemotherapy with Paclitaxel, Ifosfamide and Cisplatin (TIP) were started, followed by two autologous transplantations. Subsequently, ascites and peritoneal metastases were observed at CT scan again (Figure S1). When the metastasis firstly appeared, the patient underwent genetic counseling, where a screening of 52 cancer-related genes and a liquid biopsy approach were proposed and approved to identify germline tumor predisposing variants and somatic driver events (Supplementary Tables 1, 2). Blood test revealed the c.2393G>A (NM_000321) variant in RB1, reported as unknown in the rb1-lsdb database (http://rb1-lsdb.d-lohmann.de) with the ID: RB1_00572. Bioinformatics prediction tools for missense variants were in favor of a deleterious effect and splicing bioinformatics tools predicted the introduction of a *de novo* exonic acceptor splice site (3'SS) at position c.2394 with high confidence (Table 1). The resulting effect on the transcript was supposed to be the skipping of 69 nt at the 5' of exon 23. mRNA analysis was thus performed to confirm the prediction. Unexpectedly, *in vitro* splicing analysis, performed on blood RNA by RT-PCR, revealed the full skipping of exon 23 together with the retention of 11 nucleotides of intron 22 (r.2326_2489delins2325+1_2325+11) (Δ23▾VGQK) (Figure 2A). The resulting transcript is generated by the activation of a cryptic 5'splice site located in the intron 22 (c.2325+12) together with the usage of the 3'SS of exon 24, with the skipping of the canonical 3'SS of exon 23 (Figure 2B). This splicing alteration is predicted to result in the partial loss of the carboxy-terminal domain (C-term) with the restoration of the reading frame thanks to the insertion of four residues (p.(Pro776_Arg830delinsValGlyGlnLys)). The 3D protein model of the aberrant splicing variant showed the loss of organization of the secondary structure of the C-term and, consequently, of the complexity of the tertiary structure (Figure 2C). Sequencing of the full-length transcript revealed the presence of both A/G nucleotides at position c.2393, suggesting a “leaky” effect of the variant (Figure 2D). Densitometric analysis performed with ImageJ on the gel bands showed the aberrant spliced transcript to be ~32% of the total one, indicating that the mutant allele is still able to produce a still relevant percentage of normal spliced transcript (Figure 2E). For this reason, the impact of the missense variant on the protein structure and activity was investigated in order to understand its involvement in the protein function impairment. Six *in silico* tools (SIFT, PolyPhen-2, CADD, SNAP2, MutationTaster2, FATHMM) were queried about the impact of the missense variant on the protein function and the predictions were in favor of a deleterious effect (Table 1) (23–29). In addition, MutationTaster predicted the loss of *LIMD1* binding site. To confirm *RB1* biallelic inactivation in the tumor, NGS analysis was performed on DNA isolated from FFPE tissue slides of the ovarian cyst. A non-sense variant, c.1359T>A (p. (Tyr453^*^)), was discovered in about 15% of the analyzed molecules, percentage indicative of a contamination from DNA of the surrounding normal tissue. A liquid biopsy approach, representative of the invasive clone(s), was employed to discover driver events involved in tumor progression. Cell-free DNA isolation from plasma was performed when the patient already showed peritoneal metastases. NGS analysis of a panel of 77 cancer-related genes showed, in addition to the germline variant found in *RB1*, the presence of three pathogenic mutations in four genes: p.(Ser371Cys) in *FGFR3*, p.(Arg445^*^) in *SMAD4*, p.(Tyr205Ser) in *TP53* and p.(Lys151^*^) in *MSH2* in a percentage of 0.10, 0.15, 0.17, and 0.33, respectively.

**Table 1:** *In silico* predictions for the c.2393G>A variant.

***RB1*** **c.2393** **G>A**	**Missense prediction tools**											
	**SIFT**		**PolyPhen**		**CADD**		**SNAP2**		**FATHMM**		**MutationTaster2**	
	**Score**	**Prediction**	**Score**	**Prediction**	**Score**	**Phred**	**Score**	**Prediction**	**Score**	**Prediction**	**Score**	**Prediction**
	0	deleterious	0.99	probably damaging	4.278	32	55	effect	−1.99	damaging	43	disease causing
	**Splicing prediction tools:** ***de novo*** **3' SS in position c.2394**											
	**SpliceSiteFinder-like [0–100]**			**MaxEntScan [0–16]**			**NNSPLICE [0–1]**			**Human Splicing Finder [0–100]**		
	**WT**	**mut**		**WT**	**mut**		**WT**	**mut**		**WT**	**mut**	
	0	84.7		0	7.2		0	0.9		0	89.4	

## Discussion

The knowledge of the genetic landscape of MOGCTs is quite limited due to their rarity and heterogeneity and a general wide response to chemotherapy, often resulting in a good prognosis. However, a component of yolk sac tumor represents a poor prognosis indicator, meaning most of the time a platinum-resistant phenotype, with few therapeutic options available (30, 31). In our opinion, the study of the genetics of these rare tumors must be considered of main interest to find molecular targets and open for new therapeutic approach. In this report we showed a *RB1* genetic mutation implied in the pathogenicity of a mixed MOGCT with teratoma and yolk sac tumor component.

The c.2393G>A variant in *RB1*, found in heterozygous state in blood DNA by NGS, was further investigated to assess its impact on the protein function. A splicing impairment, leading to an in-frame deletion of part of the C-term domain and the loss of C-term organization was shown. Despite the most studied region involved in pRB function is the pocket domain, C-term has recently acquired more consideration as a regulatory domain involved in several activities. C-term has indeed the ability to bind *E2F1* in a cdk independent manner, even in a hyperphosphorylated condition. This binding persists in S phase of the cell cycle and it is responsible for the transcription of pro-apoptotic genes in response to DNA damage (32). Moreover, C-term represents the binding site for *LIMD1*, a tumor suppressor gene known to prevent Rb phosphorylation, thus promoting *E2F1* binding to the pocket domain and consequent inhibition of the cell cycle progression (33, 34). Due to these findings, the identified variant must be considered hypomorphic, partially affecting the protein function. This allowed us to hypothesize a “parent-of-origin” effect, a broadly studied mechanism in retinoblastoma. Hypomorphic variants affecting the paternal allele are more likely to predispose to RB than the ones on the maternal allele, while, maternally-inherited hypomorphic variants usually don't lead to RB development, but can predispose to other neoplasms in the adult age (14, 18, 20, 21). Here the proband was not affected by retinoblastoma in her childhood, and, as expected, the variant was inherited from the mother. We cannot exclude the proband or her mother to carry a benign retinoma, since no information about the *fundus oculi* was available.

The hypothesis of a pRB involvement in ovarian germ cell tumors predisposition is supported by the finding that *RB1* conditional knockout in ovarian germ cells lead to the development of unilateral ovarian teratoma in mice (35, 36). Interestingly, all the mice screened in the study by Yang et al. developed only unilateral teratoma, so preneoplastic lesions, implicating that besides Rb inactivation, other genes or regulatory elements must be involved to promote tumor initiation. *RB1* involvement in GCTs pathogenicity is also supported by the evidence that *RB1* is often lacking or low expressed in GCTs and by a previous report of a girl developing a primary immature teratoma following bilateral retinoblastoma, so probably carrying a *RB1* mutation (37). As biallelic *RB1* inactivation alone is responsible for a pre-neoplastic lesion (retinoma) in the retina, here it might contribute to the ovarian teratoma initiation, even if more evidence/case are needed to support this hypothesis (38). More events following *RB1* inactivation are likely needed for the aggressive yolk sac tumor development, as we found by a liquid biopsy approach, in *TP53, SMAD4, FGFR3*, and *MSH2*. *TP53* and *RB1* cooperate to repress the cell cycle in the presence of a DNA damage, so mutations in both these factors could mean a loss of cell cycle equilibrium with a consequent uncontrolled proliferation (39). Mismatch repair proteins are involved in the pathogenicity of ovarian cancer in the context of the Lynch syndrome, moreover reduced expression of *MSH2* or *MLH1* have been linked to reduced cisplatin sensitivity of testicular germ cell tumors with a differentiated teratoma component, so this mutation could partially explain the chemoresistance to cisplatin of the mature teratoma with yolk sac tumor component we reported here (40, 41). Loss of *SMAD4* is known to be associated with tumor progression and chemoresistance in different types of cancer, while alterations in *FGFR3* differently correlate with tumorigenesis and outcome in diverse carcinoma (42, 43). So, the identified somatic events could be responsible for the malignant transformation and chemotherapy resistance.

In conclusion, we identified and characterized for the first time a germline splicing variant in *RB1* in an 18-year-old patient carrying a mixed ovarian germ cell tumor with components of mature teratoma and yolk sac tumor. The variant has been linked to tumor predisposition and additional events have been found by liquid biopsy to be involved in tumor progression e aggressiveness. These results provide a first news inside the genetics of mixed ovarian germ cell tumors, despite additional cases are needed to confirm the association between *RB1* variants and OGCTs development.

## Data Availability Statement

The raw data supporting the conclusions of this article will be made available by the authors, without undue reservation, to any qualified researcher.

## Ethics Statement

Written informed consent was obtained from the next of kin for the publication of any potentially identifiable images or data included in this article.

## Author Contributions

EG performed the experiments and drafted the manuscript. CF, FV, AG, MP, and RT contributed to the experiments and data acquisition. FC, LL, AF, and MM contributed to histologic data interpretations, clinical data collections, and genetic counseling. FA and AR made substantial contributions to conception of the study and reviewed the manuscript. All authors contributed to the article and approved the submitted version.

## Conflict of Interest

The authors declare that the research was conducted in the absence of any commercial or financial relationships that could be construed as a potential conflict of interest.
