# Supplementary material for: RB1 Germline Variant Predisposing to a Rare Ovarian Germ Cell Tumor: A Case Report
Source: Front Oncol. 2020 Aug 21;10:1467. doi: 10.3389/fonc.2020.01467 (PMC7471930; doi:10.3389/fonc.2020.01467)
Supplement: Supplementary file 1 [file Data_Sheet_1.docx]

**Supplementary Appendix**

**Material and Methods**

**Sample collection and histologic diagnosis**

DNA samples were obtained at the Medical Genetics Unit of the Azienda Ospedaliera Universitaria Senese (A.O.U.S, Siena, Italy) upon the signature of informed consent for both diagnostic and research purposes. Genomic DNA was extracted from EDTA peripheral blood samples using MagCore HF16 (Diatech Lab Line, Jesi, Ancona, Italy). The ovarian cyst was formalin fixed and paraffin embedded (FFPE) and then cutted according to the usual pathological methods. The mixed ovarian germ cell tumour with teratoma and yolk sac tumour components was identified in hematoxylin–eosin‐stained sections. Sections 5 µm thick were deparaffinized, rehydrated, and stained with Mayer hematoxylin and yellow eosin, then dehydrated with xylene. Slides were observed through an inverse microscope.

**NGS on ovarian cyst tissue**

DNA from formalin-fixed paraffin embedded (FFPE) tissue section was isolated at the Pathology Unit, A.O.U.S (Siena, Italy) employing the Genomic DNA FFPE ONE-STEP kit (Diatech Labline, Jesi, Ancona, Italy) for MagCore HF16 (Diatech Lab Line, Jesi, Ancona, Italy) and then sent to the Unit of Medical Genetics, A.O.U.S (Siena, Italy). A panel including all the exons of RB1 was used for NGS analysis; library preparation, sequencing and analysis was performed as indicated above. Integrative Genomics Viewer was used to visualize the sequenced reads as previously described (Robinson, J.T.et al, *Nat. Biotechnol.* **2011**, *29*, 24–26.)

**NGS on blood DNA and variant validation**

Blood DNA was screened for variants using an amplicon-based panel covering all the coding sequence of 52 cancer related genes by NGS (coverage of 98,2% , exon padding of 25 bp) (Supplemetary table 1). Library were prepared employing the Ion AmpliSeq^TM^ Library Kit 2.0 (Life Technologies, Carls-bad, CA) and sequenced on the Ion Torrent PGM, using the Ion PGM^TM^ Sequencing 200 kit v2, according to manifacturers’ instructions. by ThermoFisher Scientific, Waltham, Massachusetts, USA). Variants were annotated using in-house software that combines information from variant-based annotation databases (ExAC, 1000-genomes, and avSNP) and the bioinformatics algorithms SIFT, CADD, MutationAssessor, and PhyloP for predicting variant pathogenicity. Variants with coverage less than 100x were filtered out. Detected variants were validated by Sanger sequencing on ABI Prism 330 genetic analyzer (PE Applied Biosystems).

**In silico predictions**

Alamut®Visual (Interactive biosoftware, Rouen, France) was employed for the *in silico* splicing prediction of the identified variant, variation of 15% in MaxEntScan and 5% in Human Splicing Finder were used as cut offs for the eligibility of the selected variant for the *in vitro* splicing analysis (Houdayer C et al, Hum Mutat. 2012 Aug;33(8):1228-38). For the missense prediction seven tools were used: SIFT PolyPhen-2, PON-P2, CADD, SNAP2, MutationTaster2, FATHMM (Vaser, R et al,. *Nat Protoc* **2016**, *11*, 1–9; Adzhubei, I.A et al, *Nat. Methods* **2010**, *7*, 248–249; Niroula, A et al, *PLoS ONE* **2015**, *10*, e0117380; Rentzsch, P et al, *Nucleic Acids Res.* **2019**, *47*, D886–D894; Bromberg, Y and Rost, B, *Nucleic Acids Res.* **2007**, *35*, 3823–3835;Schwarz, J.M. et al, *Nat. Methods* **2014**, *11*, 361–362; Shihab, H.A. et al, *Hum. Genomics* **2014**, *8*, 11).

**mRNA analysis**

Total RNA from the patient and healthy controls was then isolated from peripheral blood withdrawn in PAXgene Blood RNA Tubes (PreAnalytiX®, Qiagen, Hilden, Germany) (http:/www.qiagen.com) with the PAXgene®Blood RNA Kit (IVD) (PreAnalytiX®) following the manifacturer’ instructions. cDNAs were obtained using QuantiTect®Reverse Transcription Kit (Qiagen). Specific primers were designed (refseq: NM_000321) to detect splicing aberration by PCR: fw-5’-ATGCTGTTCAGGAGACATTC-3’, rv-3’-ACTTCTCAGAAGTCCCGAAT-5’. A no-template cDNA control, and five normal control cDNA samples were included. PCRs were performed using GoTaq DNA Polymerase reagents (5× GoTaq Flexi buffer; 25 mM MgCl2, GoTaq Hot Start Polymerase), in addition with 2 mM dNTPS, milliQ H2O and 100 ng of cDNA, for a final volume of 50 μL. Amplification was performed in a Thermal Cycler 2720 (Applied Biosystem, Foster City, CA, USA) with the following PCR program: 95°C for 5 min; 35 cycles at 95°C for 30 s, 62°C for 30 s, 72°C for 60 s; 72 ◦C for 5 min. 10 μL of amplified PCR products were mixed with the GoTaq® Flexi Reaction Buffers (Promega, [Madison, Wisconsin,](https://www.google.com/search?rlz=1C1CHWA_itIT662IT662&q=Madison+(Wisconsin)&stick=H4sIAAAAAAAAAOPgE-LUz9U3MKswKilR4gAx08qNKrW0spOt9POL0hPzMqsSSzLz81A4VhmpiSmFpYlFJalFxYtYhX0TUzKL8_MUNMIzi5Pz84oz8zQBDkGmaFkAAAA&sa=X&ved=2ahUKEwi5zIHDy-vkAhXBJ1AKHTdTDDgQmxMoATAZegQIDhAH) USA) and separated by electrophoresis on a 2% agarose gel. Bands were displayed in a UV transilluminator and the different bands were purified using the MinElute® Gel Extraction kit (Qiagen). Each PCR product was sequenced using PE Big dye terminator cycle sequencing kit on an ABI Prism 3130 Genetic Analyzer (Applied Biosystems). The software Sequencer (v.5.6) was used for sequence analysis.

**3D protein modeling**

PDB files of the FL and the aberrant spliced proteins were generated with I-TASSER and used to visualize the 3D model in EzMol (Yang, J et al, *Nat. Methods* **2015**, *12*, 7–8; Reynolds, C.R. et al, *J. Mol. Biol.* **2018**, *430*, 2244–2248).

**Cell-free DNA isolation and analysis**

The cell-free DNA was extracted from 4 ml of plasma using AVENIO ctDNA Expanded kit (Roche, Hacienda Drive Pleasanton, CA) according to manufacturer's instructions (Supplementary table 2). cfDNA quality and quantity were verified respectively using the Agilent™ High Sensitivity DNA Kit (Agilent Technologies, Palo Alto, CA) on Agilent2100 Bioanalyzer (Agilent Technologies) and Qubit™ dsDNA HS Assay Kits on Qubit 2.0 fluorometer (Invitrogen, Carlsbad, CA, USA). cfDNA sequencing was performed using AVENIO ctDNA Expanded kit (Roche, Hacienda Drive Pleasanton, CA) on Illumina NextSeq 550 (San Diego, California, USA). The sequencing analysis was performed using AVENIO Oncology Analysis Software (Roche).

**Supplementary table 1**

52 cancer-related genes included in the NGS panel

| BRCA1 | PTEN | STK11 | FANCB | RET |
| --- | --- | --- | --- | --- |
| BRCA2 | CDKN2A | ENG | FANCC | NF1 |
| RB1 | ATM | POLD1 | FANCD2 | NF2 |
| TP53 | CHEK2 | POLE | FANCE | SUFU |
| APC | BRIP1 | CDK4 | FANCF | PTCH1 |
| CDH1 | PALB2 | VHL | FANCG | PRKAR1A |
| MLH1 | BAP1 | FH | FANCI | TSC1 |
| MSH2 | DICER1 | FLCN | FANCL | TSC2 |
| MSH6 | BMPR1A | MUTYH | FANCM | RAD51C |
| PMS2 | SMAD4 | FANCA | MEN1 | RAD51D. |

**Supplementary figure 1**


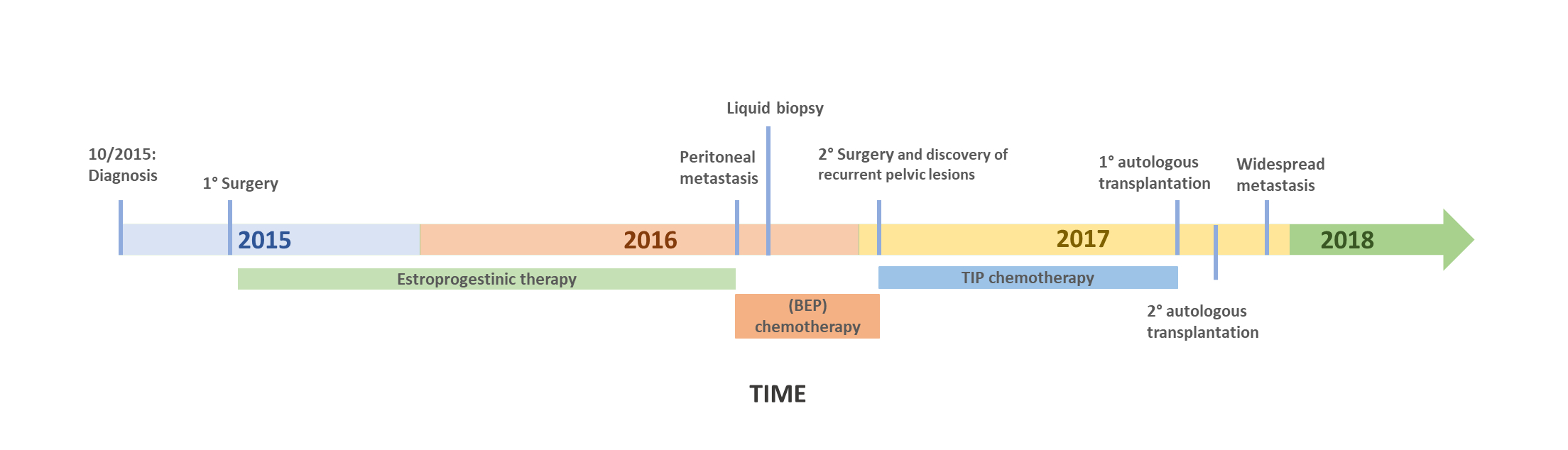


**Supplementary figure 1.** Timeline depicting the treatment history of the case report.

**Supplementary table 2**

77 cancer driver genes included in the Avenio ctDNA Expanded kit for liquid biopsy

| ABL1 | CDKN2A | GATA3 | MSH2 | RB1 |
| --- | --- | --- | --- | --- |
| AKT1 | CSF1R | GNA11 | MSH6 | RET |
| AKT1 | CTNNB1 | GNAQ | MTOR | RNF43 |
| ALK | DDR2 | GNAS | NF2 | ROS1 |
| APC | DPYD | IDH1 | NFE2L2 | SMAD4 |
| AR | EGFR | IDH2 | NRAS | SMO |
| ARAF | ERBB2 | JAK2 | NTRK1 | STK11 |
| BRAF | ESR1 | JAK3 | PDCD1LG2 | TP53 |
| BRCA1 | EZH2 | KDR | PDGFRA | TERT promoter |
| BRCA2 | FBXW7 | KEAP1 | PDGFRB | TSC1 |
| CCND1 | FGFR1 | KIT | PIK3CA | TSC2 |
| CCND2 | FGFR2 | KRAS | PIK3R1 | UGT1A1 |
| CCND3 | FGFR3 | MAP2K1 | PMS2 | VHL |
| CD274 | FLT1 | MAP2K2 | PTCH1 |  |
| CDK4 | FLT3 | MET | PTEN |  |
| CDK6 | FLT4 | MLH1 | RAF1 |  |
